# *Synechococcus elongatus* PCC 7942 as a Platform for Bioproduction of Omega-3 Fatty Acids

**DOI:** 10.3390/life12060810

**Published:** 2022-05-29

**Authors:** María Santos-Merino, Raquel Gutiérrez-Lanza, Juan Nogales, José Luis García, Fernando de la Cruz

**Affiliations:** 1 Instituto de Biomedicina y Biotecnología de Cantabria, Universidad de Cantabria—CSIC, 39011 Santander, Spain; raquel.gutierrezlanza@unican.es (R.G.-L.); fernando.cruz@unican.es (F.d.l.C.); 2 Department of Systems Biology, Centro Nacional de Biotecnología (CSIC), 28049 Madrid, Spain; jnogales@cnb.csic.es; 3 Interdisciplinary Platform for Sustainable Plastics towards a Circular Economy, Spanish National Research Council (SusPlast-CSIC), 28040 Madrid, Spain; jlgarcia@cib.csic.es; 4 Department of Microbial and Plant Biotechnology, Centro de Investigaciones Biológicas Margarita Salas (CSIC), 28040 Madrid, Spain

**Keywords:** desaturases, overexpression, *Synechococcus elongatus* PCC 7942, alpha-linolenic acid, stearidonic acid

## Abstract

Alpha-linolenic acid and stearidonic acid are precursors of omega-3 polyunsaturated fatty acids, essential nutrients in the human diet. The ability of cyanobacteria to directly convert atmospheric carbon dioxide into bio-based compounds makes them promising microbial chassis to sustainably produce omega-3 fatty acids. However, their potential in this area remains unexploited, mainly due to important gaps in our knowledge of fatty acid synthesis pathways. To gain insight into the cyanobacterial fatty acid biosynthesis pathways, we analyzed two enzymes involved in the elongation cycle, FabG and FabZ, in *Synechococcus elongatus* PCC 7942. Overexpression of these two enzymes led to an increase in C18 fatty acids, key intermediates in omega-3 fatty acid production. Nevertheless, coexpression of these enzymes with desaturases DesA and DesB from *Synechococcus* sp. PCC 7002 did not improve alpha-linolenic acid production, possibly due to their limited role in fatty acid synthesis. In any case, efficient production of stearidonic acid was not achieved by cloning DesD from *Synechocystis* sp. PCC 6803 in combination with the aforementioned DesA and DesB, reaching maximum production at 48 h post induction. According to current knowledge, this is the first report demonstrating that *S. elongatus* PCC 7942 can be used as an autotrophic chassis to produce stearidonic acid.

## 1. Introduction

Alpha-linolenic acid (ALA, 18:3n-3) and stearidonic acid (SDA, 18:4n-3) are omega-3 fatty acids (FAs) with considerable potential as dietary supplements to improve human health [1,2]. They are essential precursors of long-chain omega-3 polyunsaturated FAs, docosahexaenoic acid (DHA), and eicosapentaenoic acid (EPA). ALA and SDA are readily converted into EPA and DHA upon consumption, so they could be used as dietary sources of omega-3 FAs [3]. Many epidemiological reports have emphasized the importance of omega-3 FAs in the prevention of cardiovascular mortality due to myocardial infarction, cardiac arrest, sudden death, and stroke [4]. Furthermore, the consumption of large amounts of omega-3 is linked to a beneficial effect on the nervous system, such as a reduced risk of depression and suicide [5,6] and a delay of the neurological degeneration produced by aging [7,8].

Microbial production of omega-3 FAs gained major attention in the last years as an alternative source in commercial production [9,10]. Currently, omega-3 FAs are obtained from fish oils [11] or certain plants [12,13]. Continuous overfishing of the seas, the ever-increasing world population, as well as the limited number of plants containing significant amounts of omega-3 FAs have motivated a search for alternative sources that are both sustainable and economical. The main efforts in engineering microbial cells to produce omega-3 FAs focused on the genetic engineering of *Escherichia coli*, which demonstrated a great potential to produce high levels of these FAs [14,15,16,17,18]. However, omega-3 FA production by heterotrophic microbial cultures is expensive, mostly due to the high costs of the carbon sources, accounting for 60–75% of total expenses [10,19]. Microalgae and cyanobacteria are promising candidates for omega-3 FA production due to their ability to convert atmospheric carbon dioxide into FAs [10]. Moreover, the culture of these photosynthetic organisms does not compete with arable land use [20] and can use wastewater as a culture medium [21]. A series of advantages make cyanobacteria a preferred organism to produce FAs rather than eukaryotic microalgae, including a simpler genetic background that eases manipulation and the availability of genetic tools that allow cyanobacterial genetic engineering [20].

**Figure 1:**
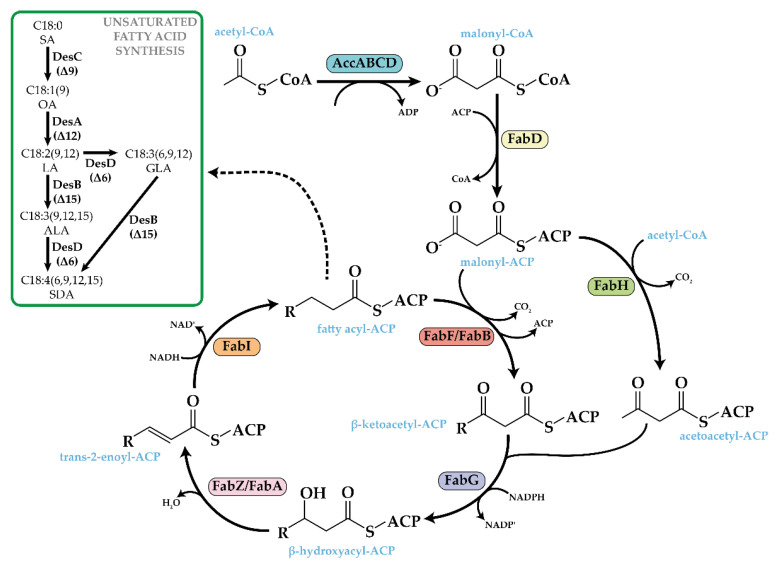
Omega-3 FA synthesis pathway in cyanobacteria. AccABCD (acetyl-CoA carboxylase) catalyzes the formation of malonyl-CoA using two molecules of acetyl-CoA. The malonyl moiety is transferred to the acyl-carrier protein (ACP) by FabD (malonyl-CoA ACP transacylase. The initial condensation reaction is catalyzed by FabH (β-ketoacyl-ACP synthase III), reducing acetyl-CoA and malonyl-ACP to form acetoacetyl-ACP. AccABCD, FabD, and FabH are enzymes that perform reactions in the initiation step. The next reaction in the elongation cycle is catalyzed by FabG (β-ketoacyl-ACP reductase), which reduces acetoacetyl-ACP to form β-hydroxyacil-ACP, which is dehydrated by FabZ/FabA (β-hydroxyacyl-ACP dehydratase) to produce *trans*-2-enoyl-ACP. The final step in the cycle is catalyzed by FabI (*trans*-2-enoyl-ACP reductase). The resulting fatty acyl-ACP can be elongated further by FabF/FabB (β-ketoacyl-ACP synthase II/I) or if the fatty acyl-ACP contains 16 or 18 carbon atoms (C16:0-ACP or C18:0-ACP, respectively) can be unsaturated by the action of desaturases. FabF, FabG, FabZ, and FabI are enzymes that perform reactions in the elongation cycle. In the desaturation step, four consecutive desaturases (DesC or Δ9, DesA or Δ12, DesB or Δ15, and DesD or Δ6) catalyzed the conversion of stearic acid (SA) into stearidonic acid (SDA). OA, oleic acid; LA, linoleic acid; GLA, γ-linolenic acid; ALA, α-linolenic acid.

Short-chain omega-3 FAs, ALA, and SDA are naturally synthesized in cyanobacteria [22]. Their biosynthetic pathway involves several complex reaction steps, including initiation, elongation, and desaturation enzymatic activities (Figure 1) [10]. Cyanobacteria accumulate omega-3 FAs in their thylakoid membranes, especially in response to cold [23] and salt stress conditions [24], albeit not large amounts of omega-3 FAs are accumulated. Thus, different approaches have been used to boost the production of omega-3 FAs in cyanobacteria. The main strategy consists of the overexpression of endogenous and exogenous desaturases and enzymes involved in the introduction of a double bond in a specific position of long-chain FAs (Figure 1) [21,25,26,27,28]. Modification of culture conditions, such as increasing the light intensity or/and decreasing the temperature during cyanobacterial culture, have been successfully used to improve omega-3 FA yields [29,30,31,32]. In addition, our group has previously demonstrated that omega-3 FA levels may be also increased by targeting some FA biosynthesis (fab) genes (Figure 1) [33]. In a recent report, the expression of the vesicle-inducing protein in plastids (Vipp1), a thylakoid membrane formation enhancer, led to high yields of omega-3 FAs in several cyanobacterial species [34].

The limited information available about the function and regulation of fab genes [35] complicates engineering efforts to improve FA production in cyanobacteria. To address this issue, this work identifies the function of two enzymes (FabG and FabZ) involved in the elongation cycle of the FA synthesis pathway (Figure 1) by overexpressing the corresponding genes. FA elongation represents the major pathway defining the chain length of saturated and unsaturated FAs and, consequently, determining the yield of omega-3 FAs in cells. Moreover, the overexpression of endogenous *fabG* and *fabZ* in combination with DesAB desaturases of *Synechococcus* sp. PCC 7002 (hereafter, Ss7002) in ALA production was investigated, showing a negative impact on the accumulation of omega-3 FAs in *Synechococcus elongatus* PCC 7942 (hereafter, Se7942). On the other hand, SDA was successfully produced in Se7942 when using desaturase DesD of *Synechocystis* sp. PCC 6803 (hereafter, Ss6803). We establish that longer post-induction times were necessary to obtain higher yields of SDA.

## 2. Materials and Methods

### 2.1. Strains and Cultivation Conditions

All cyanobacterial strains used in this study were cultivated at 30 °C by bubbling 3% CO_2_ under continuous light at 60 µmol photons m^−2^ s^−1^ in BG11 medium [36] supplemented with 10 mM NaHCO_3_. The antibiotics used for selecting cyanobacterial transformants were neomycin at 5 or 25 µg/mL (Neo5 or Neo25), spectinomycin at 10 or 20 µg/mL (Sp10 or Sp20) and chloramphenicol at 5 or 10 µg/mL (Cm5 or Cm10). The strains used are depicted in Supplementary Table S1.

*E. coli* DH5α cells were employed for transformation with recombinant plasmids. Bacterial cultures were grown on Luria-Bertani (LB) medium at 37 °C at 150 rpm. When required, LB was supplemented with antibiotics: spectinomycin at 100 µg/mL (Sp100), ampicillin at 100 µg/mL (Ap100), kanamycin at 50 µg/mL (Kn50), and chloramphenicol at 25 µg/mL (Cm25).

### 2.2. Plasmid Constructions

All plasmids used in this work are described in Supplementary Table S2. Primers used to clone genes and verify the mutant strains are listed in Supplementary Tables S3 and S4, respectively. All constructions were checked by PCR and DNA sequencing.

Gene encoding the Δ6 desaturase (D6D) from Ss6803, *desD*, and *trc* promoter were synthesized by ATG:biosynthetics (Merzhausen, Germany) after codon optimization for Se7942 expression. The 3-ketoacyl-ACP reductase and 3-hydroxyacyl-ACP dehydratase genes *fabG* and *fabZ* were amplified from the genomic DNA of Se7942 colonies. To overexpress the desaturase gene *desD* of Ss6803 (*sll0262*; GenBank Accession Number: NC_020286), plasmid pMSM278 was generated. For its construction, the *trc* promoter and *desD* gene were excised from the pGH-D6D vector (provided by ATG:biosynthetics, Merzhausen, Germany) at the KpnI and NotI sites and cloned in the same sites of the pMSM253 vector [33]. To overexpress the *fabG* gene of Se7942 (Synpcc7942_0684; GenBank Accession Number: NC_007604) in two neutral chromosomal regions, NS1 and NS2, plasmids pMSM310 and pMSM317 were constructed. Gene *fabG* was amplified with primer pair 380–381, digested with EcoRI and AvaI, and ligated to the same sites of pUAGC280, producing plasmid pMSM310. To generate pMSM317, the P*trc::fabG* fragment was amplified from pMSM310 plasmid DNA using primers 364 and 384. The amplicon was digested with KpnI/NotI and cloned in the same sites of pMSM249, rendering plasmid pMSM317. We performed the construction of pMSM319 to co-overexpress *fabF* and *fabG* genes of Se7942 (Synpcc7942_0537 and Synpcc7942_0684; GenBank Accession Number: NC_007604) in the neutral chromosomal region, NS2. To generate pMSM319, the P*trc::fabG* fragment was amplified from pMSM310 plasmid DNA using primers 364 and 384. The amplicon was digested with KpnI/NotI and cloned in the same sites of pMSM253, rendering plasmid pMSM319. For overexpressing the Se7942 *fabZ* gene (Synpcc7942_0930; GenBank Accession Number: NC_007604), the coding sequence was amplified by PCR using primers 382 and 383, digested with EcoRI and BamHI, and ligated to the same sites of pUAGC280 to produce pMSM311. We performed the construction of pMSM321 to co-overexpress *fabF* and *fabZ* genes of Se7942 (Synpcc7942_0537 and Synpcc7942_0930; GenBank Accession Number: NC_007604) in the neutral chromosomal region, NS2. To generate pMSM321, the P*trc::fabZ* fragment was amplified from pMSM311 plasmid DNA using primers 385 and 386. The amplicon was digested with XbaI/XhoI and cloned in the same sites of pMSM253, rendering plasmid pMSM321.

### 2.3. Natural Transformation of Se7942

Transformation of Se7942 was performed according to a protocol described earlier [37]. Briefly, cyanobacterial strains were transformed by incubating cultures at OD_720_ ≤ 0.5 with 500 ng plasmid DNA for 24 h in the dark at 30 °C. Cell-DNA mixed cultures were spread on nitrocellulose filters (Millipore) on BG11 plates and incubated for 24 h at 30 °C with continuous light. BG11 plates supplemented with appropriate antibiotics were used for selecting recombinant strains. Mutant segregation was achieved by repeatedly transferring individual transformant colonies to fresh selective plates. PCR was used to verify chromosomal integration of targets, followed by DNA sequencing using oligonucleotides listed in Supplementary Tables S2 and S4.

### 2.4. Culture of Engineered Cyanobacterial Strains for Fatty Acid Production

Liquid cultures of Se7942 were grown at 30 °C and 3% CO_2_ in tubes containing 60 mL of BG11 without antibiotics in a Multi-Cultivator MC 1000-OD (Photon Systems Instruments, Drasov, Czech Republic) using cool white light (60 µmol photons m^−2^ s^−1^). Cultures were inoculated at an initial OD_720_ ~ 0.05. The temperature was shifted to 22 °C when cultures reached OD_720_ ~ 0.5–0.6, and 1 mM IPTG (to induce *trc* promoter) was added. Samples were taken 24 h post induction unless indicated otherwise.

### 2.5. Fatty Acid Analysis

Cyanobacterial FA composition was carried out by gas chromatography flame ionization detection (GC-FID) analysis using pelleted cells. Preparation of FA methyl esters (FAMEs) was performed as previously described [38]. Briefly, 60 mL of cyanobacterial cultures were pelleted by centrifugation at 4500× *g* for 30 min at 4 °C. The supernatant was removed, and the cell pellets were frozen at −80 °C for storage and to facilitate cell lysis. FA methyl esters (FAMEs) were prepared as previously described [38]. Saponification of FAMEs was conducted by adding 1 mL of saponification reagent (45 g NaOH, 150 mL methanol, 150 mL deionized H_2_O), vortexing for 10 s, heating the samples for 5 min at 100 °C, vortexing for 10 s, and heating again for 25 min. Samples were then rapidly cooled to room temperature. Methylation was performed by adding 2 mL of methylation reagent (325 mL 6 N HCl, 275 mL methanol), vortexing for 10 s, and heating for 10 min at 80 °C. After cooling to room temperature, FAMEs were removed from the acidic aqueous phase and transferred to an organic phase by adding 1.25 mL extraction solvent (hexane:methyl tert-butyl ether (MTBE) 1:1), mixing in a laboratory rotator for 10 min, and removing the lower aqueous phase. The organic phase was washed in 3 mL base wash (10.8 g NaOH, 900 mL distilled water) for 5 min with end-over-end mixing. Two-thirds of the upper solvent phase was removed for FAME analysis, and the rest was stored at −20 °C. Concentrated FAMEs were analyzed on a gas chromatograph (model 7890A, Agilent) equipped with a capillary column DB-23 (Agilent Technologies, Santa Clara, CA, USA) and a flame ionization detector using helium as a carrier gas at 52.248 psi constant pressure. The initial oven temperature was set at 130 °C and programmed up to 215 °C at a rate of 2.75 °C/min and up to 230 °C at a rate of 40 °C/min. The injector temperature was set at 270 °C, and the FID detector at 280 °C. FAMEs were identified by comparing the retention time with the commercial standard Supelco^®^ 37 component FAME mix (Sigma Aldrich, St. Louis, MO, USA).

### 2.6. Statistical Analysis

All presented results are representative of at least three replicate experiments. As appropriate for the experimental design, data are reported as the mean value + SD. Statistical analyses were conducted using GraphPad Prism 8 (GraphPad Software Inc., San Diego, CA, USA) and Minitab (MiniTab Inc., State College, PA, USA) software. Comparisons between multiple groups were made using one-way analysis of variance (ANOVA) followed by Tukey’s multiple comparison test or by an unpaired Student’s *t*-test. A minimal level of statistical significance for differences in values was considered to be *p* < 0.05.

## 3. Results

### 3.1. Modification of the Lipid Profile of Se7942 by Overproduction of Enzymes Involved in the Elongation Cycle of the Fatty Acid Synthesis Pathway

FabG, a β-ketoacyl-ACP reductase, catalyzes the first of the two reduction steps in the elongation cycle of the FA synthesis pathway (Figure 1). In this reversible reaction, β-ketoacyl-ACP is reduced to β-hydroxyacyl-ACP at the concomitant expense of NADPH [39]. Homologous expression of the *fabG* gene in *E. coli* increased the content of C16:0 and C18:0 by 2- or 3-fold, respectively [40].

**Figure 2:**
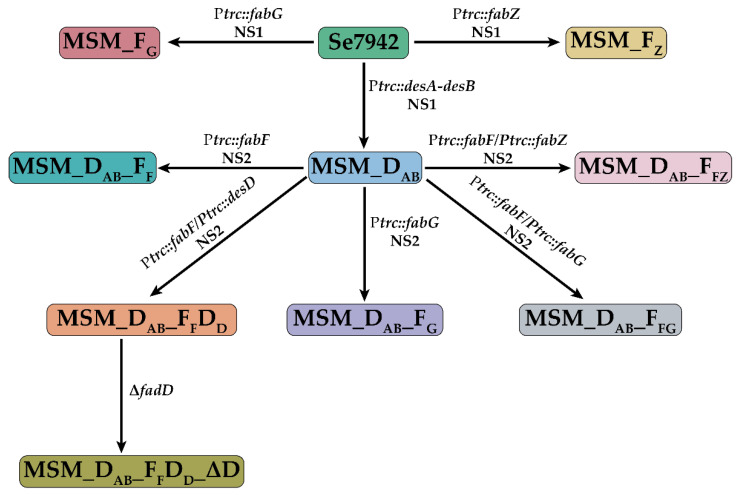
Genealogy of sequential modifications performed in Se7942 for omega-3 production. The complete description of each strain is shown in Supplementary Table S1.

**Figure 3:**
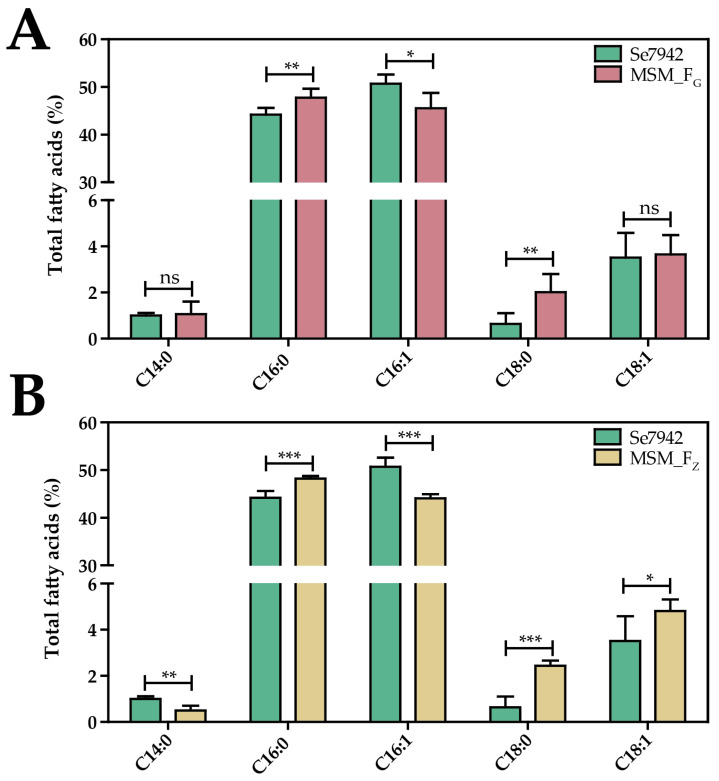
Effect of *fabG* and *fabZ* overexpression on FA composition. (**A**) FA content of wt Se7942 (green bars) and the *fabG* mutant derivative, MSM_F_G_ (red bars). (**B**) FA content of wt Se7942 (green bars) and the *fabZ* mutant derivative, MSM_F_Z_ (yellow bars). In both graphs, the different FA species are shown along the *x*-axis. The *y*-axis shows the percentage of each FA with respect to the total amount of FAs analyzed. Data are the average of at least three independent biological replicates and are represented as the mean + SD. ns (not significant), *p* > 0.05; * *p* < 0.05, ** *p* < 0.01, *** *p* < 0.001, by unpaired Student’s *t*-test.

To test the effect of *fabG* overexpression in Se7942, MSM_F_G_ strain harboring P*trc::fabG* cassette installed in neutral site 1 (NS1) was constructed (Figure 2). The *fabG* overexpression led to an increase in the content of C18:0 and C16:0 FAs and a decrease in C16:1 FA (Figure 3A). In *E. coli*, the reduction in the C16:1 level was not observed when fabG was overexpressed, whereas an increase in C18:0 was also detected [40]. Our results demonstrate that although the regulation of FA synthesis is different in these two bacteria, *fabG* overexpression led to similar changes in the FA profile in both organisms.

The next reaction in the FA elongation cycle is catalyzed by FabZ, a β-hydroxyacyl-ACP dehydratase (Figure 1), which performs the dehydration of β-hydroxyacyl-ACP to *trans*-2-enoyl-ACP [41]. In *E. coli*, two genes codify for two different β-hydroxyacyl-ACP dehydratases, *fabA* and *fabZ*. In addition to its dehydration activity, FabA has a second function related to the isomerization of *trans*-2-decenoyl-ACP into *cis*-3-decenoyl-ACP, which is the first reaction toward the synthesis of unsaturated FAs [42]. Many cyanobacterial species lack a recognizable FabA homolog since the unsaturated FA synthesis is performed by desaturases [43]. The homologous overexpression of the *fabZ* gene in *E. coli* resulted in an about 2-fold increase in C16:0 and C18:0 levels [40]. The activity of FabZ is inhibited by high levels of (p)ppGpp in this organism [44,45], but no regulators have been described for FabZ in cyanobacteria.

To analyze the function of FabZ in Se7942, this gene was overexpressed using *trc* promoter in NS1 of Se7942 in the strain MSM_F_Z_. This mutant showed a significant decrease in C14:0 and C16:1 FAs and a significant increase in C16:0, C18:0, and C18:1 FAs (Figure 3B). These results indicate that high levels of FabZ activity improved the transformation of C14:0 and C16:1 into C18 species. Moreover, FabZ in Se7942 seems to perform a similar function to the same enzyme in *E. coli* since the FA profile was similar when FabZ activity was increased [40].

### 3.2. Overproduction of FabG or FabZ Has a Detrimental Effect in the Production of Alpha-Linolenic Acid in Se7942

In cyanobacteria, three desaturation reactions convert oleic acid (OA) into ALA, catalyzed by DesC (Δ9 desaturase), DesA (Δ12 desaturase), and DesB (Δ15 desaturase) [46] (Figure 1). Se7942 only possesses the *desC* gene and lacks the remainders [22], not naturally producing omega-3 FA. We have previously demonstrated that the overexpression of *desA* and *desB* genes from Ss7002 in Se7942 led to ALA production [33]. In addition, ALA yield was increased by *fabF* overexpression and *fadD* deletion, modifications that increased C18:0 FA levels. As we showed above, the individual overexpression of *fabG* and *fabZ* also led to increases in C18:0 species. Thus, we hypothesized that the combined overexpression of *fabG* and *fabZ* with *desAB* might lead to an improvement in ALA yield. Therefore, considering these assumptions, we decided to co-overexpress *fabG*, *fabZ,* and *desAB* desaturase genes.

**Figure 4:**
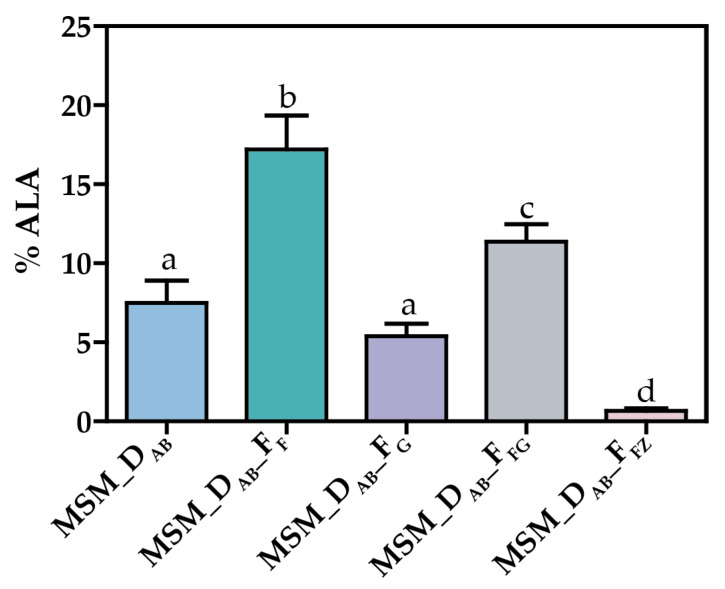
ALA production in Se7942 derivative strains containing the overexpression of *fabF*, *fabG,* and *fabZ* genes. Data are the average of at least three independent biological replicates and are represented as the mean + SD. Significance was calculated by one-way ANOVA followed by Tukey’s multiple comparison test. Bars labeled with different letters are significantly different (*p* < 0.05).

To examine the effect of *fabG* overexpression in ALA synthesis, MSM_D_AB__F_G_ and MSM_D_AB__F_FG_ overproducing strains were developed (Figure 2). The effect of *fabG* overexpression in ALA production was compared with two control strains: MSM_D_AB_ and MSM_D_AB__F_F_ (Figure 4). Both strains are able to produce ALA since they express DesAB, but MSM_D_AB__F_F_ produces a large amount due to the positive effect of *fabF* [33]. Contrary to our expectations, we found that the combined overexpression of *fabG* with *desA* and *desB* in MSM_D_AB__F_G_ strain did not lead to an increase in ALA levels, suggesting that FabG overproduction is detrimental to ALA production. In the same way, when *fabF*, *fabG*, *desA,* and *desB* genes were combined in the strain MSM_D_AB__F_FG_, higher levels of ALA were obtained but still lower than the ones found in MSM_D_AB__F_F_. It might indicate that the positive effect due to *fabF* overexpression was largely diminished by the overexpression of *fabG*. Interestingly, MSM_D_AB__F_FG_ showed a decrease in C16:0 contents and an increase in C18:0 levels compared to MSM_D_AB__F_G_ (Table 1). The increase in C18:0 could be caused for the overexpression of *fabG* since this change was also detected in MSM_D_AB__F_G_. On the other hand, the decrease in C16:0 was only detected in MSM_D_AB__F_FG_, suggesting that this change is due to the effect of *fabF* overexpression, as previously described [33]. Taking all these results together, we suggest that *fabG* overexpression has a negative effect on ALA production in Se7942.

Following the same strategy, the MSM_D_AB__F_FZ_ strain was developed to test the effect of *fabZ* overexpression in ALA synthesis (Figure 2). This strain co-overexpresses *fabZ* and *fabF* genes from NS2. The co-overexpression of *fabZ*, *fabF,* and desaturase genes (*desA* and *desB*) did not lead to an increase in ALA levels (Figure 4). In fact, the amount of ALA produced by MSM_D_AB__F_FZ_ was the lowest reported in all strains analyzed in this work (0.66%) (Figure 4). This result indicates that the overexpression of the *fabZ* gene, as in the case of *fabG*, has a negative effect on ALA production. Similar to the MSM_D_AB__F_FG_ strain, the MSM_D_AB__F_FZ_ strain showed a decrease in C16:0 FA content and an increase in C18:0 FA levels compared to the MSM_D_AB__F_F_ strain, and an increase in C18:1 FA level was not detected in MSM_D_AB__F_FG_ (Table 1).

### 3.3. Overproduction of FabF and DesABD in a Se7942 Derivative Strain Leads to High Stearidonic Acid Yields

**Figure 5:**
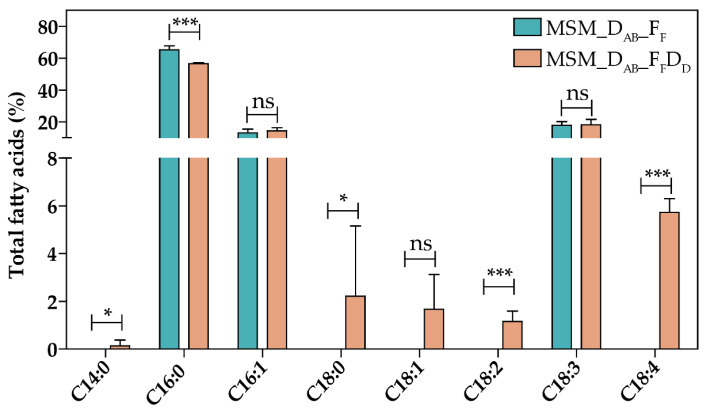
SDA production in Se7942. Effect of ALA production in the FA Profile. MSM_D_AB__F_F_ and MSM_D_AB__F_F_D_D_, ALA mutant producers, are represented by green and orange bars, respectively. Data are the average of at least three independent biological replicates and are represented as the mean + SD. ns (not significant), *p* > 0.05, * *p* < 0.05, *** *p* < 0.001, by unpaired Student’s *t*-test.

To produce SDA in Se7942, three desaturases need to be heterologously expressed: DesA (Δ12 desaturase), DesB (Δ15 desaturase), and DesD (Δ6 desaturase) [46] (Figure 1). To test the ability of Se7942 to produce SDA, we used a previously engineered ALA-producer strain expressing *desA* and *desB* genes from Ss7002 [33], in which we expressed the *desD* gene from Ss6803 generating MSM_D_AB__F_F_D_D_ that should be able to transform ALA into SDA (Figure 2). As expected, these modifications led to the production of SDA up to 5.73% (Figure 5). In addition, other changes were observed in the FA profile of MSM_D_AB__F_F_D_D_ in comparison to the profile shown by MSM_D_AB__F_F_ as follows (i) a significant reduction in C16:0 FA, suggesting that this FA is used in the production of SDA; (ii) a significant rise in C14:0, C18:0, and C18:2 FAs, indicating that elongation reactions are highly active in MSM_D_AB__F_F_D_D_; (iii) an absence of γ-linolenic acid (GLA), another product of DesD activity can be used to produce SDA; (iv) no statistical changes in C18:3 (ALA) levels between both strains, indicating that DesD has greater affinity to linoleic acid (LA) than to ALA as substrate.

Our previous results demonstrated that *fadD* deletion improves ALA production in Se7942 [33]. To test if this deletion might have a beneficial effect on SDA production, this modification was introduced in the MSM_D_AB__F_F_D_D_ strain, generating MSM_D_AB__F_F_D_D__ΔD (Figure 2). The amounts of SDA decreased significantly in the new strain, albeit no other significant changes were observed in the FA profile of this strain (Table 2). These results might indicate that *fadD* deletion has a detrimental effect on SDA production, but the nature of this negative effect is unclear.

### 3.4. Stearidonic Acid Production Is Improved by Increasing the Induction Time of the Genes Involved in Its Synthesis

**Figure 6:**
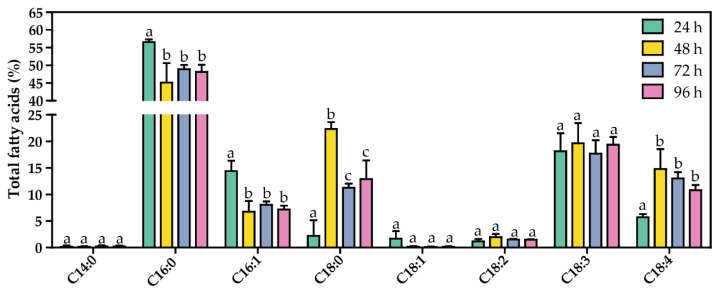
FA profile of MSM_D_AB__F_F_D_D_ strain at different post-induction times. Data are the average of at least three independent biological replicates and are represented as the mean + SD. Significance was calculated by one-way ANOVA followed by Tukey’s multiple comparison test. Bars labeled with different letters are significantly different (*p* < 0.05).

Since the synthetic pathway for SDA production implemented in Se7942 requires the overproduction of several enzymes, we hypothesized that longer post-induction times could yield increased production of SDA. To test this hypothesis, we analyzed the SDA produced at various times after induction (i.e., 24 h, 48 h, 72 h, and 96 h). Interestingly, SDA levels were significantly higher at 48 h post induction compared to 24 h (14.8% vs. 5.7%, respectively) (Figure 6). Higher post-induction times (>48 h) did not increase SDA production. In addition, cells presented significantly lower levels of C16:0 and C16:1 FAs. A significant increase in C18:0 was detected at 48 h post induction, coinciding with the induction time where SDA reached its highest level but was not maintained after this time point (Figure 6). Thus, we can suggest that SDA production stimulates two main changes in the FA profile: (i) a decrease in C16:0 and C16:1 FAs and (ii) an increase in C18:0 levels. SDA can be produced through DesD desaturation of ALA or through DesB desaturation of GLA (Figure 1). Since GLA was not detected and ALA levels did not change at any post-induction times (Figure 6), we hypothesize that SDA is produced by the desaturation of GLA by DesB rather than the desaturation of ALA by DesD.

## 4. Discussion

Cyanobacteria are promising microorganisms for sustainable bioproduction since they directly convert CO_2_ and light into fuels, chemicals, and other value-added products [20]. Moreover, these organisms naturally synthesize omega-3 FAs with different degrees of unsaturation, depending on the expression of the desaturase enzymes involved in the FA synthesis pathway. Biosynthesis of omega-3 FAs has been thoroughly studied in three models of unicellular cyanobacteria, Ss6803 [32,47,48,49,50], Ss7002 [29,30,31,47], and *Synechococcus* sp. NKBG 15041c [51]. Four desaturases are involved in the synthesis of omega-3 FAs in cyanobacteria: DesC (Δ9 desaturase), DesA (Δ12 desaturase), DesB (Δ15 or ω-3 desaturase), and DesD (Δ6 desaturase) (Figure 1). In addition, it was demonstrated that only four cyanobacterial species naturally produce SDA (Ss6803, *Tolypothrix tenuis*, *Lyngbya* sp. PCC 8106, and *Nodularia spumigena* [22]). Nevertheless, only in Ss6803, the activity of the enzymes was characterized [32,47,48,49,50]. Se7942 does not naturally produce omega-3 FAs since it lacks these three desaturases, although it was previously engineered with desaturases from Ss6803 [47] and from Ss7002 [33] to produce variable yields of ALA. To our knowledge, SDA production has not been reported in Se7942. In addition, there is little information available related to the FA synthesis and regulation in cyanobacteria in general, making it difficult to achieve higher levels of production.

Overexpression of bottleneck enzymes and deletion of competing pathways are the most common strategies used to increase the production of valuable compounds in cyanobacteria [44]. The ALA production yield was improved in Se7942 by overexpressing FabF, an enzyme that participates in the cycle of FA elongation, and by removing the activity of FadD, involved in the competitive FA degradation pathway [33]. Both modifications have in common that result in an increase in C18 species, as we report in this work with *fabG* and *fabZ* overexpression. However, these new genetic constructs did not improve ALA synthesis (Figure 4 and Table 1). Whereas *fabF* overexpression and *fadD* deletion have in common a pronounced increase in C18:1 [33], this raise was not observed when *fabG* and *fabZ* were overexpressed (Figure 3). These results suggest that higher levels of C18:1 are required for an improvement in omega-3 FA production. In fact, a previously published report concluded that FabF, mainly involved in the extension of C16:1 into C18:1, is the key-limiting reaction in the synthesis of unsaturated fatty acids in this cyanobacterium [52]. In addition, SDA only improved when FabF was overexpressed (Figure 5 and Table 2), but not when FadD was eliminated (Table 2). In Ss6803, *fadD* deletion results in down-regulation of DesD protein expression, but it did not have any effect on DesA and DesB protein levels [53]. Although we are expressing DesD from Ss6803 in Se7942, we speculate that this unknown mechanism of regulation could be maintained between cyanobacterial species. Taken together, our results indicate that the first step in the elongation reaction catalyzed by FabF seems to determine the ability of the cells to produce omega-3 FAs in Se7942. In addition, the reactions of reduction and dehydration catalyzed by FabG and FabZ, respectively, seem to have less influence on the biosynthesis of FAs.

Production of SDA by overexpressing desaturases was previously used as a strategy in several cyanobacterial species, reaching SDA levels up to 12% of the total FA content in Ss7002 [20] and up to 13% of total FAs in Ss6803 [19]. Here, we report SDA levels up to 5.7% at 24 h post induction, increasing to 14.8% at 48 h (Figure 5 and Figure 6). To reach these levels, besides overexpression of desaturases, we manipulated the FA synthesis pathway by modifying the dosage of what looks like the limiting step in Se7942, the FabF reaction. Above 48 h post induction, SDA yields did not further improve, suggesting that we could have reached the maximum level of SDA that can be accumulated in Se7942 membranes. In a recent publication, SDA levels higher than the above were obtained using a new strategy: overproduction of Vipp1 (vesicle-inducing protein in plastids) [34]. These authors reported SDA yields of 10%, 17%, and 27% of total lipids in *Anabaena* sp. PCC 7120, Ss7002, and *Leptolyngbya* sp. strain BL0902, respectively. In cyanobacteria, Vipp1 promotes thylakoid membrane biogenesis and maintenance [54], and its overexpression in Ss6803 increases the thylakoid membrane abundance [55]. It is tempting to speculate that Vipp1 overexpression may allow an additional increase in SDA production in the strains described in this work by increasing the number of thylakoid membranes where omega-3 FAs could be accumulated.

In cyanobacteria, DesD, also known as Δ6 desaturase, is the enzyme that catalyzes the addition of a double bond at the 6th carbon–carbon bond position in FAs [56]. This enzyme is required for the conversion of ALA into SDA or LA into GLA (Figure 1) [57]. In human cells, it is well established that ALA is the preferred substrate for Δ6 desaturase, and its affinity for ALA is 2–3 times greater than for LA [58]. In Ss6803, on the contrary, Δ6 desaturase displays a higher affinity for LA than for ALA, favoring the production of GLA [57], as demonstrated by heterologously expressing DesD in Ss7002 [27] in *Anabaena* sp. PCC 7120 [57] or in Se7942 (by also coexpressing DesA) [57]. In our experiments, we did not detect GLA when SDA was produced by DesABD coexpression (Figure 6), supporting the idea that Ss6803 DesD has a higher affinity for LA rather than for ALA. Taken together, our data suggest that synthesis of SDA in Se7942 is accomplished by Δ15-desaturation of GLA, not by Δ6-desaturation of ALA. Thus, we propose that the sequence of desaturation reactions to produce SDA in Se7942 would be SA → OA → LA → GLA → SDA (catalyzed by DesC, DesA, DesD, and DesB, respectively) (Figure 1).

## 5. Conclusions

In this study, the effects of the FabG and FabZ enzymes involved in the elongation step of the FA synthesis pathway in Se7942 were examined by overexpressing the corresponding genes and analyzing the resulting FA profiles. Although the genetically engineered strains increased the C18 FA levels, which in principle should lead to an increase in ALA production, unexpectedly, they did not. Combined overexpression of *desA* and *desB* from Ss7002, *desD* from Ss6803, and *fabF* from Se7942, on the other hand, was a useful strategy to produce SDA in Se7942, which was maximal at long post-induction times. This work establishes a new strategy for improving the production of SDA in cyanobacteria

## Figures and Tables

**Table 1:** FA profile of ALA-producer strains.

FA (%) ^1^	MSM_D_AB_	MSM_D_AB__F_F_	MSM_D_AB__F_G_	MSM_D_AB__F_FG_	MSM_D_AB__F_FZ_
C14:0	1.56 ± 0.40 ^a^	0 ± 0 ^b^	1.03 ± 0.15 ^c^	0.53 ± 0.06 ^d^	0.82 ± 0.05 ^c^
C16:0	70.44 ± 4.14 ^a^	65.19 ± 2.58 ^a^	69.17 ± 6.52 ^a^	57.68 ± 1.16 ^b^	50.46 ± 1.50 ^b^
C16:1	16.22 ± 2.95 ^a^	13.04 ± 2.43 ^a^	16.4 ± 1.41 ^a^	14.90 ± 1.81 ^a^	42.33 ± 2.16 ^b^
C18:0	2.73 ± 0.47 ^a^	0 ± 0 ^b^	4.94 ± 0.63 ^c^	13.65 ± 0.93 ^d^	2.46 ± 0.52 ^a^
C18:1	0 ± 0 ^a^	0 ± 0 ^a^	0.06 ± 0.03 ^a^	0.07 ± 0.07 ^a^	3.29 ± 0.28 ^b^
C18:2	0 ± 0 ^a^	0 ± 0 ^a^	0.28 ± 0.06 ^a^	0.60 ± 0.30 ^b^	0 ± 0 ^a^
C18:3	7.49 ± 1.43 ^a^	17.9 ± 2.18 ^b^	4.99 ± 1.20 ^a^	11.58 ± 1.12 ^c^	0.64 ± 0.18 ^c^

^1^ Expressed as mean ± standard deviation. Significance was calculated by one-way ANOVA followed by Tukey’s multiple comparison test. Values for each FA labeled with different letters are significantly different (*p* < 0.05).

**Table 2:** FA profile of SDA-producer strains.

FA (%) ^1^	MSM_D_AB__F_F_D_D_	MSM_D_AB__F_F_D_D__ΔD
C14:0	0.14 ± 0.24	0.71 ± 0.39
C16:0	56.57 ± 0.73	55.65 ± 5.82
C16:1	14.39 ± 1.96	18.29 ± 4.07
C18:0	2.22 ± 0.94	0.71 ± 0.78
C18:1	1.67 ± 1.45	3.38 ± 1.03
C18:2	1.16 ± 0.44	0.95 ± 0.76
C18:3	18.12 ± 3.40	19.40 ± 4.26
C18:4	5.73 ± 0.58	0.92 ± 1.08 ***

^1^ Expressed as mean ± standard deviation. Significance was calculated by unpaired Student’s *t*-test, *** *p* < 0.001.

## Data Availability

Not applicable.

## Supplementary Materials

The following supporting information can be downloaded at: https://www.mdpi.com/article/10.3390/life12060810/s1, Table S1. List of cyanobacterial strains used in this study, Table S2. Plasmids used in this study, Table S3. Oligonucleotides used to clone genes in this study, Table S4. Oligonucleotides used to verify mutants in this study. References [33,59,60] are cited in the Supplementary Materials.
