# Supplementary material for: Synechococcus elongatus PCC 7942 as a Platform for Bioproduction of Omega-3 Fatty Acids
Source: Life (Basel). 2022 May 29;12(6):810. doi: 10.3390/life12060810 (PMC9224711; doi:10.3390/life12060810)
Supplement: Supplementary file 1 [file life-12-00810-s001.zip › life-1741479-supplementary.pdf]

Table S1. List of cyanobacterial strains used in this study.

**Strains**	**Relevant characteristics**[1]	**References**[2]
Synechococcus elongatus PCC 7942	Wild type	PCC
GRPS1	*S. elongatus* PCC 7942 with mutation R43 in *rps12* gene; Sm^r	[59]
MSM_D$_{AB}$	*S. elongatus* PCC 7942 transformed with pMSM201 encoding P*trc*::*desA-desB* (NS1); Sp^r	[33]
MSM_D$_{AB}$_F$_{F}$	MSM_AB transformed with pMSM253 encoding P*trc*::*fabF* (NS2); Sp^r Cm^r	This work
MSM_D$_{AB}$_F$_{F}$D$_{D}$	MSM_AB transformed with pMSM278 encoding P*trc*::*fabF*/P*trc*::*desD* (NS2); Sp^r Cm^r	This work
MSM_D$_{AB}$_F$_{F}$D$_{D}$_ΔD	GRPS1 transformed with pMSM201 encoding P*trc*::*desA-desB* (NS1), pMSM278 encoding P*trc*::*fabF*/P*trc*::*desD* (NS2) and PMSM_AB6 to delete *fadD*; Sp^r Cm^r Neo^r	This work
MSM_F$_{G}$	*S. elongatus* PCC 7942 transformed with pMSM310 encoding P*trc*::*fabG* (NS1); Sp^r	This work
MSM_F$_{Z}$	*S. elongatus* PCC 7942 transformed with pMSM311 encoding P*trc*::*fabZ* (NS1); Sp^r	This work
MSM_D$_{AB}$_F$_{G}$	MSM_AB transformed with pMSM317 encoding P*trc*::*fabG* (NS2); Sp^r Cm^r	This work
MSM_D$_{AB}$_F$_{FG}$	MSM_AB transformed with pMSM319 encoding P*trc*::*fabF*/P*trc*::*fabG* (NS2); Sp^r Cm^r	This work
MSM_D$_{AB}$_F$_{FZ}$	MSM_AB transformed with pMSM319 encoding P*trc*::*fabF*/P*trc*::*fabZ* (NS2); Sp^r Cm^r	This work

[1] NS1, neutral site 1; NS2, neutral site 2. Constructions integrated in NS1 and NS2 were verified by PCR using primer pairs 102-103 and 106-108, respectively. Sm^r, streptomycin resistance; Sp^r, spectinomycin resistance; Cm^r, chloramphenicol resistance; Neo^r, neomycin resistance. Plasmids mentioned in this table are listed in Supplementary Table S1.

[2] PCC, Pasteur Culture Collection.

Table S2 Plasmids used in this study.

Plasmid	Relevant genotype[1]	Source or reference	Primers to check plasmids[2]
pUAGC280	NS1 targeting vector with *lacI*^{q} and P*trc*; Ap^{r} Sm^{r}	[60]	
pGH-D6D	pGH::(P*trc-desD*); Ap^{r}	ATG:biosynthetics	
pMSM201	pUAGC280::(*desA-desB*); Sp^{r}	[33]	
pMSM249	pAM1580::Δ*luxAB*; Cm^{r} Ap^{r}	[33]	
pMSM253	pMSM249::(P*trc-fabF*); Cm^{r} Ap^{r}	[33]	
pMSM266	pMSM1::USDT-*fadD*; Km^{r} Cm^{r}	[33]	
pMSM278	pMSM253::(P*trc-desD*); Cm^{r} Ap^{r}	This work	106-282
pMSM310	pUAGC280::*fabG*; Sp^{r}	This work	104-105
pMSM311	pUAGC280::*fabZ*; Sp^{r}	This work	104-105
pMSM317	pMSM249::(P*trc-fabG*); Cm^{r} Ap^{r}	This work	106-282
pMSM319	pMSM253::(P*trc-fabG*); Cm^{r} Ap^{r}	This work	106-282
pMSM321	pMSM253::(P*trc-fabZ*); Cm^{r} Ap^{r}	This work	397-398

[1] Ap^{r}, ampicillin resistance; Sm^{r}, streptomycin resistance; Sp^{r}, spectinomycin resistance; Cm^{r}, chloramphenicol resistance.

[2] Primer pairs used to check the constructions by PCR and DNA sequencing. The sequence of these primers is detailed in Supplementary Table S3.

Table S3. Oligonucleotides used to clone genes in this study.

Number	Sequence (5′ → 3′)[1]
364	CCTAGGGTACC**TTGACAATTAATCATCCGGCTCG**
380	ACCATGGAATTC**ATGACTGCTTTGCCCCTAACCG**
381	TCTAGACTCGAG**CTAGGCCATCACCAAGCCGC**
382	ACCATGGAATTC**ATGACCGTCAACCCGGATGC**
383	TCTAGAGGATCC**TTACTCCACGAGCGAGAACAACAGTTC**
384	TGTTGCGGCCGC**CTAGGCCATCACCAAGCCGC**

[1] Sequences hybridizing to the template DNA are in bold. Restriction sites are underlined.

Table S4. Oligonucleotides used to verify mutants in this study.

Number	Sequence (5′ → 3′)
102	GCTTGCCTTCCTATGGTTCGG
103	CCTGTGCAGCAGGAGCGG
104	GTGGACCGCTTGCTGCAACTC
105	GCTTGGCAGACCGCTGGTG
106	CGCTTCCCACGCTGAGAGG
108	TGCTGGGTAGTTCTCCGCTGC
282	CGACTGTGAATGAACGTCGCTTG
108	TGCTGGGTAGTTCTCCGCTGC
397	CAAGCGACGTTCATTCACAGTCG
398	AACGCTGGTGAAAGTAAAAGATGCTG
